# Impact of Walking and Respiratory Training on Cardiopulmonary Function and Activity Endurance in Patients With Chronic Heart Failure

**DOI:** 10.1002/clc.70123

**Published:** 2025-03-30

**Authors:** Qiu‐Chen Wang, Jiang‐Ying Li, Xiao‐Su Ni, Wen‐Wen Zhao, Xiao‐Cui Wang, Li‐Chun Wang

**Affiliations:** ^1^ Department of Cardiology The Affiliated Taizhou People's Hospital of Nanjing Medical University Taizhou China

**Keywords:** activity endurance, cardiac function, chronic heart failure, pulmonary function, respiratory training, walking exercise

## Abstract

**Objective:**

This study aimed to examine the impact of combining walking with respiratory training in patients with chronic heart failure (CHF).

**Methods:**

Eighty patients with CHF, admitted to the Department of Cardiology at the Affiliated Taizhou People's Hospital of Nanjing Medical University between January 2024 and June 2024, were selected as study participants. They were randomly assigned to either a control group or an observation group, with 40 patients in each group. The control group received standard rehabilitation care, while the observation group underwent walking combined with respiratory training along with standard rehabilitation care. Cardiac function, pulmonary function, and activity tolerance were compared between the two groups.

**Results:**

After 3 months of intervention, the observation group exhibited lower levels of heart rate (HR), left ventricular end‐diastolic diameter (LVEDD), and left ventricular end‐systolic diameter (LVESD) compared to the control group, while the left ventricular ejection fraction (LVEF) was significantly higher (*p* < 0.05). Additionally, the forced vital capacity (FVC), forced expiratory volume in 1 s (FEV_1_), FEV_1_/FVC ratio, and 6‐min walking distance were greater in the observation group compared to the control group (*p* < 0.05).

**Conclusion:**

In patients with CHF, combining walking and respiratory training significantly enhances cardiopulmonary function and activity tolerance, demonstrating potential clinical use.

## Graphical abstract

This study investigates the impact of combined walking and respiratory training on cardiopulmonary function and activity endurance in patients with chronic heart failure (CHF). Eighty CHF patients were randomly assigned to either a control group receiving standard rehabilitation or an observation group undergoing walking and respiratory training in addition to standard care. After a 3‐month intervention, the observation group exhibited significant improvements in cardiac function (lower heart rate, left ventricular end‐diastolic diameter, and left ventricular end‐systolic diameter; higher left ventricular ejection fraction), pulmonary function (higher forced vital capacity, forced expiratory volume in 1 s, and FEV1/FVC ratio), and activity endurance (longer 6‐min walking distance). These findings suggest that combining walking with respiratory training enhances cardiopulmonary function and activity tolerance in CHF patients, offering potential clinical benefits.

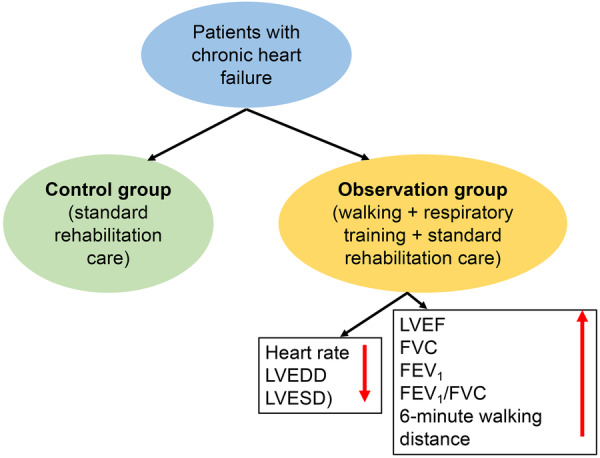

**Abbreviations**
6MWT: 6‐min walk test
CHF: chronic heart failure
FEV1: forced expiratory volume in 1 s
FVC: forced vital capacity
HR: heart rate
LVEDD: left ventricular end‐diastolic diameter
LVEF: left ventricular ejection fraction
LVESD: left ventricular end systolic‐dimension
NYHA: New York Heart Association Functional Classification
RPE: rating of perceived exertion

## 1 Introduction

Chronic heart failure (CHF) is a chronic cardiovascular condition characterized by myocardial cell damage or loss, excessive hemodynamic stress, diastolic dysfunction, and cardiac remodeling, with key clinical manifestations including dyspnea and fluid retention [1, 2]. Management of CHF typically involves pharmacological treatments aimed at controlling disease progression, along with basic interventions like disease monitoring, health education, and cardiac rehabilitation activities. These measures are used to mitigate the advancement of the condition. In recent years, with global population aging and enhanced survival rates for cardiovascular diseases, the prevalence of CHF has been steadily rising. Aerobic exercise has become an integral component of comprehensive treatment strategies for CHF, and Wu Jiangbo has demonstrated that appropriate aerobic exercise can reduce oxidative stress, enhance myocardial contractility, enhance muscle oxidative capacity, and promote better cardiopulmonary function and activity tolerance in patients [3]. Among aerobic exercises, walking, due to its simplicity and safety, has been widely adopted in clinical settings [4]. However, patients with CHF often experience respiratory discomfort during physical activity due to the disruption in the normal coordination between neural inspiratory drive and the dynamic response of the respiratory system. As a result, walking by itself may not fully address the training needs of patients with CHF [5]. Given this, respiratory training along with walking exercise has been proposed as a more comprehensive rehabilitation approach for CHF. Respiratory training encompasses specific breathing techniques like abdominal breathing and pursed‐lip breathing, which aim to strengthen respiratory muscles, enhance lung ventilation efficiency, and enhance gas exchange, ultimately preventing reductions in exercise tolerance [6]. Despite its potential, there have been limited studies that have clarified the mechanisms underlying the effects of combining walking exercises with respiratory training in patients with CHF. Therefore, this study conducts a detailed analysis of the impact of this combined intervention on patients with CHF through controlled trials, assessing cardiopulmonary function and activity tolerance indicators, so as to provide a foundational reference for future research.

## 2 Data and Methods

### 2.1 General Data

Patients with CHF who were treated at the Affiliated Taizhou People's Hospital of Nanjing Medical University from January 2024 to June 2024 were selected as research participants. The sample size was calculated using the formula: *N* = *P*(1−*P*)/[*α* ^2^/*Z* ^2^ + *P*(1−*P*)/*E*] (*p* = 0.5, *α* = 0.05, *Z* = 1.109, *E* = 104) resulting in a total of 80 patients. The 80 eligible patients were assigned numeric identifiers from 1 to 80, and random non‐repeated group sampling was conducted using an electronic computer, with a sampling ratio of 1:1. Inclusion criteria were as follows: (1) Patients met the diagnostic criteria for CHF as outlined in the Chinese Guidelines for the Diagnosis and Treatment of Heart Failure 2024, confirmed by echocardiography, cardiac function measurements, myocardial biopsy, and other examinations [7]. Specifically, the diagnosis of CHF was made based on medical history, clinical presentation, and auxiliary tests. The major manifestations include shortness of breath, palpitations, fatigue, ankle swelling, elevated jugular venous pressure, a displaced apical impulse, peripheral edema, and orthopnea. Electrocardiogram results reveal abnormalities such as atrial fibrillation, Q waves, or left ventricular hypertrophy. Chest X‐ray scans indicate pulmonary congestion or edema. Elevated natriuretic peptide levels and echocardiography, which assesses left ventricular ejection fraction (LVEF), confirm the diagnosis of CHF. (2) Age > 18 years; (3) Patients classified as New York Heart Association Functional Classification (NYHA) grade II to III [8]; (4) Patients with stable vital signs; (5) Patients who voluntarily consented to participate in the study. Exclusion criteria were: (1) Individuals unable to participate in rehabilitation activities; (2) Individuals with impaired functions of other organs; (3) Individuals with a history of psychiatric disorders; (4) Individuals with limb dysfunction; (5) Patients experiencing unstable angina pectoris or myocardial infarction within the past month. No significant differences were observed in general data between the two groups (*p* > 0.05) (Table 1). Informed consent was obtained from all patients, and the study was approved by the Ethics Committee of the Affiliated Taizhou People's Hospital of Nanjing Medical University.

**Table 1:** General data regarding the two groups of patients [*n*(%)/$\bar{x}\pm s$].

Groups	Sex		Age (years)	Duration of disease (years)	NYHA grade	
	Male	Female			Grade Ⅱ	Grade Ⅲ
Control group (*n* = 40)	23 (57.50)	17 (42.50)	57.33 ± 2.43	2.18 ± 0.38	22 (55.00)	18 (45.00)
Observation group (*n* = 40)	19 (47.50)	21 (52.50)	56.78 ± 2.42	2.08 ± 0.53	25 (62.50)	15 (37.50)
*χ* ^2^/*t* value	0.802		1.014	0.970	0.464	
*p* value	0.370		0.314	0.335	0.496	

### 2.2 Research Methods

#### 2.2.1 Control Group

Routine rehabilitation nursing was administered, with patient observation conducted over a 3‐month period. Basic examinations were conducted on the day of admission, including echocardiography, electrocardiogram stress tests, and coronary computed tomography (CT), to assess the patient's condition. Concurrently, the attending physician provided health education, detailing the fundamental causes of CHF, relevant prognostic treatments, precautions, and other essential information. Additionally, the responsible nurse offered health guidance for 5 min every 3 days, focusing on daily diet and lifestyle modifications. For patients exhibiting negative emotions, strategies such as sharing successful case experiences and providing verbal encouragement were used to alleviate distress.

Based on the New York Heart Association (NYHA) classification, corresponding cardiac rehabilitation activities were implemented. For patients with relatively stable vital signs, referring to body temperature, pulse, respiration, and blood pressure within the normal range with minimal fluctuations, classified as NYHA grade II, slow walking exercises were conducted daily, with a minimum distance of 500 m. Patients were also encouraged to perform activities of daily living, such as eating, dressing, and personal hygiene, independently. For those classified as NYHA grade III, basic exercises, including daily breathing exercises, bedside standing, and balance training, were performed for at least 5–10 min each day. Following discharge, the contact numbers of the patients were retained for follow‐up purposes. Posthospital follow‐up visits were scheduled every 15 days to monitor the conditions of the patients. Family members were instructed to closely observe the daily activities of the patients, and if symptoms like increased blood pressure, chest pain, or difficulty breathing arose, timely re‐examination and treatment were recommended.

#### 2.2.2 Observation Group

The routine nursing measures and observation duration for this group were aligned with those of the control group. Additionally, a walking exercise program combined with respiratory training was implemented, using the following specific methods:

1. Basic assessment: Before initiating the training, cardiologists conducted a comprehensive assessment of the individual circumstances, including age, disease severity, and comorbidities of each patient. This assessment informed the development of a personalized training plan, determining the appropriate exercise intensity and type for each patient. Health education was also provided to patients and their families, highlighting the purpose, benefits, and significance of walking and respiratory training to encourage active patient participation.
2. Execution methods: (1) Walking exercise: In the initial phase (intervention 1–7 days), short‐distance walking training was conducted once daily for 5–10 min. Exercise intensity was monitored using a heart rate monitor or the Rating of Perceived Exertion (RPE) scale, with gradual increases in walking distance and duration in subsequent phases. (2) Respiratory training: This included techniques such as abdominal breathing, pursed lip breathing (inhaling and exhaling through pinched nostrils or pursed lips), and breathing coordination training (practicing deep and slow breathing while walking). Training sessions were held once daily for 10–20 min. (3) Combined training: Respiratory exercises lasting 5 min were performed following 5 min of walking. Both the duration and speed of walking were progressively increased as the physical fitness of the patient improved, with corresponding adjustments in the complexity of the respiratory exercises.
3. Dynamic assessment: Every 15 days, a structured assessment and measurement method was used to dynamically assess the cardiopulmonary function and activity tolerance of the patients. Adjustments to the training plan were made based on the evaluation results.
4. Family supervision: The families of the patients were instructed to participate in the rehabilitation training and to meticulously document the implementation of the training activities, reporting these to the responsible nurse on a monthly basis. Additionally, the hospital organized health lectures to reiterate the importance of rehabilitation exercises, correct exercise techniques, and dietary and lifestyle advice. Patients were also encouraged to engage in regular follow‐up assessments.

### 2.3 Observation Indicators

#### 2.3.1 Cardiac Function

Through in‐hospital observation and follow‐up assessments, bedside echocardiography was used to detect and compare several cardiopulmonary parameters between the two patient groups. These parameters included HR (normal reference range: 60–100 beats per minute), LVEDD (normal reference range: 35–50 mm), LVESD (normal reference range: 23–40 mm), and LVEF (normal reference range: 50%–70%). Measurements were taken on the day of enrollment and after a 3‐month intervention period.

#### 2.3.2 Pulmonary Function

Through in‐hospital observation and follow‐up assessments, the forced vital capacity (FVC), forced expiratory volume in 1 s (FEV_1_), and the FEV_1_/FVC ratio were measured and compared between the two groups both on the day of enrollment and after a 3‐month intervention period. FVC Measurement: Patients were instructed to inhale deeply and then exhale forcefully to empty the lungs completely, followed by a slow inhalation to maximum volume. This process was repeated 3–5 times, and the highest value obtained was recorded as FVC. FEV_1_ Measurement: Patients were instructed to take a deep breath and then exhale forcefully for at least 4 s. This procedure was repeated 3–5 times, with the highest value recorded as FEV_1_. FEV_1_/FVC: The FEV_1_/FVC ratio was calculated by dividing the FEV_1_ value by the FVC value. A higher ratio indicates better ventilatory function. The results of these tests were systematically recorded and analyzed for comparison.

#### 2.3.3 Activity Tolerance

Through in‐hospital observation and follow‐up assessments, the walking distance was assessed using a 6‐min walking test conducted on the day of enrollment and after a 3‐month intervention period. The activity tolerance of both groups was compared based on these measurements [9]. Experimental Methodology: A 30‐m straight line was marked on flat ground, with a chair placed at each end to serve as markers and rest stations. Patients were instructed to walk back and forth along the straight line at their maximum speed for the duration of the 6‐min walking test. The total walking distance covered during this time was recorded for analysis.

### 2.4 Statistical Analysis

Statistical analysis was conducted using SPSS version 25.0. Categorical data were presented as *n* (%), and comparisons between groups were made using the *χ* ^2^ test. Continuous data were expressed as mean ± standard deviation (SD), with *t*‐tests conducted for comparisons both within and between groups. *p* < 0.05 was considered statistically significant.

## 3 Results

### 3.1 Comparison of Cardiac Function Levels Between the Two Groups

On the day of enrollment, no significant differences were observed in the cardiac function levels between the two groups (*p* > 0.05). After a 3‐month intervention, the observation group exhibited significantly lower levels of HR, LVEDD, and LVESD compared to the control group. Conversely, the LVEF in the observation group was significantly higher than that in the control group (*p* < 0.05) (Table 2).

**Table 2:** Comparison of cardiac function levels between the two groups ($\bar{x}\pm s$).

Groups	*n*	HR (times/min)		LVEDD (mm)		LVESD (mm)		LVEF (%)	
		On the day of enrollment	Following 3 months of intervention	On the day of enrollment	Following 3 months of intervention	On the day of enrollment	Following 3 months of intervention	On the day of enrollment	Following 3 months of intervention
Control group	40	89.43 ± 6.59	70.68 ± 3.34*	67.33 ± 3.47	54.03 ± 3.09*	55.48 ± 2.06	40.78 ± 1.80*	42.81 ± 4.05	58.37 ± 5.22*
Observation group	40	88.50 ± 6.56	68.96 ± 2.90*	67.70 ± 3.43	52.55 ± 2.84*	54.85 ± 2.08	38.68 ± 1.72*	42.77 ± 4.90	61.86 ± 5.33*
*t* value		0.633	2.459	0.480	2.230	1.361	5.335	0.040	2.959
*p* value		0.529	0.016	0.633	0.029	0.177	< 0.001	0.968	0.004

*Note:* Compared with that on the day of enrollment.

* *p* < 0.05.

### 3.2 Comparison of Pulmonary Function Levels Between the Two Groups

On the day of enrollment, no significant differences were noted in the pulmonary function indexes between the two groups (*p* > 0.05). Following a 3‐month intervention, the observation group demonstrated significantly higher levels of FVC, FEV_1_, and the FEV_1_/FVC ratio compared to the control group (*p* < 0.05) (Table 3).

**Table 3:** Comparing the pulmonary function indexes of the two groups ($\bar{x}\pm s$).

Groups	*n*	FVC (L)		FEV_1_ (%)		FEV_1_/FVC	
		On the day of enrollment	Following 3 months of intervention	On the day of enrollment	Following 3 months of intervention	On the day of enrollment	Following 3 months of intervention
Control group	40	3.63 ± 1.28	4.29 ± 1.32	3.22 ± 1.11	3.71 ± 1.48	0.89 ± 0.21	0.86 ± 0.23
Observation group	40	3.60 ± 1.50	4.97 ± 1.26	3.17 ± 1.97	4.85 ± 1.82	0.88 ± 0.19	0.98 ± 0.23
*t* value		0.096	2.357	0.140	3.074	0.223	2.333
*p* value		0.924	0.021	0.889	0.003	0.824	0.022

### 3.3 Comparison of Activity Tolerance Between the Two Groups

On the day of enrollment, no significant difference was observed in the walking distance measured by the 6‐min walking test between the two groups (*p* > 0.05). After 3 months of intervention, the walking distance recorded in the observation group was significantly greater than that in the control group (*p* < 0.05) (Table 4).

**Table 4:** Comparison of activity tolerance levels between the two groups ($\bar{x}\pm s$, m).

Groups	*n*	On the day of enrollment	Following 3 months of intervention
Control group	40	282.92 ± 34.93	354.54 ± 15.62*
Observation group	40	286.83 ± 36.61	366.17 ± 26.62*
*t* value		0.489	2.383
*p* value		0.626	0.020

*Note:* Compared with that on the day of enrollment.

* *p* < 0.05.

## 4 Discussion

In recent years, the increasing prevalence of modern aging has resulted in persistently high incidence and mortality rates associated with CHF, establishing it as a significant global public health concern. This condition is primarily characterized by the impaired pumping function of the heart, which leads to inadequate blood supply to bodily tissues and organs, manifesting in a range of clinical symptoms, including dyspnea, fatigue, and edema. Furthermore, as the disease progresses, patients experience a gradual decline in cardiopulmonary function and activity tolerance, severely impacting their quality of life. In the comprehensive management of CHF, nonpharmacological therapies have assumed an increasingly pivotal role. Among these, walking is a valuable adjunct to rehabilitation training, holding considerable clinical significance in cardiac rehabilitation [10]. Nevertheless, it is noted that high‐intensity walking exercise may induce an abnormal elevation in blood pressure. However, when this exercise is combined with respiratory training and the Valsalva maneuver is avoided, the risk of cardiovascular events may be mitigated.

This study demonstrated that combining walking with respiratory training effectively enhances cardiopulmonary function in patients with CHF. These findings align with the research of Cui Rong, who similarly reported improvements in cardiopulmonary function through this integrated intervention model, indicating its clinical relevance [11]. The underlying mechanisms influencing these outcomes can be elucidated. In patients with CHF, diminished cardiac pumping ability often results in inadequate energy supply to cardiomyocytes [12, 13]. Frequent walking promotes blood circulation and increases myocardial blood flow, thereby delivering larger amounts of oxygen and nutrients to the heart. This enhancement in energy metabolism within myocardial cells enhances the efficiency of cardiac function and facilitates left ventricular remodeling [14]. Additionally, walking exercise has a beneficial impact on vascular endothelial function [15]. Endothelial cells, integral components of the vascular wall, secrete various bioactive substances that regulate vascular tone. Impairment of endothelial function in patients with CHF can lead to reduced vasodilation capacity and increased vascular resistance.

However, walking can increase blood flow shear forces, stimulating endothelial cells to release relaxation factors such as nitric oxide. This response enhances cellular oxidase activity, enhances vascular endothelial function, decreases vascular resistance, increases cardiac preload, and supports the heart's pumping efficiency [16]. Furthermore, the integration of respiratory training bolsters the strength and endurance of respiratory muscles, enhances lung capacity, and enhances gas exchange efficiency, thereby alleviating pulmonary congestion and mitigating dyspnea symptoms [17]. Techniques like abdominal breathing stimulate the vagus nerve, increasing its excitability, which contributes to a reduction in heart rate and blood pressure, ultimately lowering the cardiac workload. This approach helps avoid complications like shortness of breath and hypoxia during walking, leading to a comprehensive improvement in the cardiopulmonary function of affected patients. In this study, the observed increases in FVC, FEV_1_, and the FEV_1_/FVC ratio in the observation group compared to the control group substantiate these conclusions.

Furthermore, this study revealed that combining walking and respiratory training significantly enhances the activity tolerance of patients with CHF. The rationale for this improvement lies in the fact that just walking may not sufficiently alleviate symptoms in certain patients with CHF, particularly those experiencing dyspnea. Consequently, the integration of respiratory training is clinically relevant. Respiratory training encompasses techniques like abdominal breathing and pursed lip breathing, which are designed to optimize breathing patterns and increase the strength and endurance of respiratory muscles. These techniques can effectively reduce the respiratory rate and decrease the work of breathing, enabling patients to engage in physical activities for extended periods while expending less energy. In clinical practice, walking is initiated at low intensity, with gradual increments in duration and distance to prevent overexertion. Respiratory training can be implemented as both a preparatory and relaxation component before and after each exercise session. This approach helps patients in better regulating their breathing rhythm, minimizes discomfort during physical activity, and ultimately leads to a comprehensive enhancement of activity tolerance.

Respiratory training is beneficial for heart failure patients with both reduced and preserved ejection fraction, helping to improve breathing function and quality of life. For HF patients with reduced ejection fraction, their cardiac systolic function is severely impaired. Reduced cardiac output leads to pulmonary congestion and gas exchange disorders, resulting in symptoms like dyspnea. Respiratory training strengthens respiratory muscles, improves lung ventilation, increases lung capacity and oxygen intake, reduces residual volume, accelerates blood flow, and enhances oxygen exchange, thereby alleviating dyspnea. Additionally, it helps reduce sympathetic nervous system excitability, increase muscle blood flow, improve exercise tolerance, and enhance overall quality of life. For HF patients with preserved ejection fraction, despite relatively normal systolic function, diastolic dysfunction leads to elevated left ventricular filling pressure, pulmonary congestion, and respiratory difficulties. Respiratory training can help these patients optimize breathing patterns, strengthen respiratory muscles, and improve lung compliance, thereby relieving dyspnea. It may also have positive effects on vascular endothelial function and inflammation, contributing to better cardiac function and prognosis. As a nonpharmacological treatment, respiratory training is suitable for heart failure patients across different ejection fraction ranges. Regardless of ejection fraction, this study demonstrates its effectiveness in improving respiratory function and quality of life. Furthermore, since it requires no special equipment or facilities, is simple to perform, and easy for patients to learn, respiratory training has high clinical applicability and potential for widespread implementation.

This study has certain limitations. The relatively small sample size constrains the statistical power and generalizability of the findings. Additionally, differences in the specifics of the protocols across different studies were not addressed, which complicates the comparison and comprehensive analysis of results. Furthermore, the observation period in this study was limited, leaving the long‐term effects of the intervention unexamined. To address these limitations, future research should focus on conducting large‐scale, multicenter randomized controlled trials. Such studies would provide a more robust verification of the effects of combined walking exercise and respiratory training on the long‐term prognosis of patients with CHF, thereby providing improved guidance for clinical practice.

## 5 Conclusion

In conclusion, walking along with respiratory training demonstrates a significant positive impact on improving both cardiopulmonary function and activity tolerance in patients with CHF.

## Author Contributions

**Qiu‐Chen Wang, Jiang‐Ying Li,** and **Li‐Chun Wang:** conception and design of the research. **Li‐Chun Wang, Wen‐Wen Zhao,** and **Xiao‐Cui Wang:** acquisition of data. **Jiang‐Ying Li** and **Xiao‐Su Ni:** analysis and interpretation of the data. **Xiao‐Su Ni, Wen‐Wen Zhao,** and **Xiao‐Cui Wang:** statistical analysis. **Qiu‐Chen Wang:** obtaining financing. **Qiu‐Chen Wang** and **Wen‐wen Zhao:** writing of the manuscript. **Xiao‐Cui Wang, Qiu‐Chen Wang,** and **Li‐Chun Wang:** critical revision of the manuscript for intellectual content. All authors read and approved the final draft.

## Ethics Statement

The study was conducted in accordance with the Declaration of Helsinki (as was revised in 2013). The study was approved by Ethics Committee of the Affiliated Taizhou People's Hospital of Nanjing Medical University (No.2024‐081‐01).

## Consent

Written informed consent was obtained from all participants.

## Conflicts of Interest

The authors declare no conflicts of interest.

## Data Availability

The data that support the findings of this study are available from the corresponding author upon reasonable request.
